# DNA barcoding of rumen flukes (Paramphistomidae) from bovines in Germany and Austria

**DOI:** 10.1007/s00436-021-07344-z

**Published:** 2021-10-18

**Authors:** Sandra Wiedermann, Josef Harl, Hans-Peter Fuehrer, Sandra Mayr, Juliane Schmid, Barbara Hinney, Steffen Rehbein

**Affiliations:** 1 Institute of Parasitology, Department of Pathobiology, University of Veterinary Medicine Vienna, 1210 Vienna, Austria; 2 Institute of Pathology, Department of Pathobiology, University of Veterinary Medicine Vienna, 1210 Vienna, Austria; 3 Boehringer Ingelheim Vetmedica GmbH, Kathrinenhof Research Center, Walchenseestr. 8-12, 83101 Rohrdorf, Germany

**Keywords:** Paramphistomosis, *Calicophoron*, *Paramphistomum*, DNA barcoding, Rumen fluke

## Abstract

Rumen flukes have received growing veterinary attention in western and central Europe during the past two decades because of an increase in prevalence of infection in cattle and sheep, including cases of severe clinical disease. Historically, rumen fluke infections in Europe were assumed to be caused mainly by *Paramphistomum cervi* (or species, which were later considered to be synonymous with *P. cervi*), but more recently molecular studies demonstrated *Calicophoron daubneyi* to be the predominating species. For the present investigation, adult rumen flukes isolated from 23 cattle originating from ten farms in Germany (Saxony [1], Baden-Württemberg [4], Bavaria [5]) and one farm in Austria (Tyrol) were analyzed to establish partial sequences of the mitochondrial cytochrome c oxidase subunit I (*COI*) and the complete sequence of the nuclear internal transcribed spacer 2 (*ITS2*). Flukes of five animals (dairy cows from three farms in Bavaria) were determined as *P. leydeni*, and flukes of 18 animals (dairy cows or cattle from cow-calf operations from eight farms in Saxony [1], Baden-Württemberg [4], Bavaria [2], and Tyrol [1]) were identified as *C. daubneyi*. Based on the molecular analysis of adult rumen flukes collected from cattle, the results of this investigation confirm the common occurrence of *C. daubneyi* in Germany and reveal the first definitive findings of *P. leydeni* in Germany and *C. daubneyi* in Austria.

## Introduction

Although known for several centuries to parasitize the reticulo-rumen of ruminants, the digenetic trematodes of the family Paramphistomidae have not received much attention for their parasitism in the northern hemisphere until the recent past. Despite having been reported frequently in Europe, rumen fluke infections were considered apparently as quite uncommon and regarded as of little or no clinical significance (Forbes 2021). The increasing number of reports from continental Europe, the British Isles, and Ireland over the last two decades indicates a growing prevalence of paramphistome infections in domestic ruminants. In this context, outbreaks of disease (acute intestinal paramphistomosis) in both cattle and sheep have been reported from France, the UK, and Ireland (Huson et al. 2017; Wenzel et al. 2019; Forbes 2021).

While the diagnosis of rumen fluke infection is based on gross inspection of the forestomachs for adult flukes, the detection of rumen fluke eggs in feces, or on the recovery of immature flukes in feces or at necropsy, the identification of flukes to species level is challenging (Forbes 2021). The morphological differences, which require fine-structural examination (light and electron microscopy), are quite subtle and prone to be affected by the conditions when flukes were collected. Thus, species identity and taxonomy of the paramphistomes encountered in the infection of ruminants, intermediate host specificity and life cycle, have been a matter of intense debate in the 1970s and early 1980s and resulted in several instances in confusion, misidentification, and generation of synonyms (Odening and Gräfner 1979; Sey 1980, 1982; Odening 1983). However, work over the past 15 years has shown that these problems can be addressed using molecular identification methods (Mitchell et al. 2021).

As reviewed by Wenzel et al. (2019), the rate of bovine rumen fluke infection apparently increased in the recent past in Germany, while diagnoses of paramphistomosis in Austria are rare (Hinney, unpublished). Detailed histomorphological examination of rumen flukes in Germany in the 1970s including intermediate host experimental infection studies indicated the occurrence of *Paramphistomum cervi*, *P. ichikawei*, and *Calicophoron daubneyi* in cattle (Kraneburg 1977; Odening et al. 1978). In addition, Eduardo (1982) described the presence of *P. leydeni* in cattle from Germany. The latter species was considered synonymous with *P. cervi* by Odening et al. (1978) and Odening (1983). Reviews of parasites of wild ruminants in Austria only list *P. cervi* as parasite of red deer, roe deer, and mouflon (Kutzer and Hinaidy 1969; Prosl 1973) and, based on fluke histology, records of *P. cervi* and *P. leydeni* from red deer (Eduardo 1982).

Given the limited knowledge on the paramphistome species parasitizing ruminants in both Germany and Austria, flukes collected in the year 2020 when surveying cattle for endoparasites were analyzed using molecular methods to add information on the species identity of rumen flukes from cattle in the two countries.

## Material and methods

For the present molecular study, 35 rumen flukes isolated from 23 cattle were used. The cattle (15 dairy cows, and seven young cattle plus one cow from cow-calf operations) originated from eleven pasture-based farms in Germany (ten farms: Saxony/East Saxony, one farm/five dairy cows; Baden-Württemberg/Black Forest, four cow-calf operation farms/one to three animals per farm; Bavaria/Upper Bavaria, one to three dairy cows from four farms and one animal from one cow-calf operation farm) and Austria (Tyrol, county Kitzbühel/two dairy cows from one farm), and total fluke counts were established by examination of the forestomachs.

Before the analyses, flukes were stored in physiological saline solution or 70% ethanol. DNA was extracted from the flukes with a High Pure PCR Template Preparation Kit (Roche Diagnostics GmbH, Germany) according to the manufacturer’s instructions. Two gene regions were amplified by PCR, a partial sequence of the *COI* (barcode region), as well as the complete *ITS2* sequence.

The *ITS2* and the flanking 5.8 s and 28 s regions of rDNA were amplified using amphistosome-specific primers (Amph_fwd 5′- TGT GTC GAT GAA GAG CGC AG -3′ and Amph_rev 5′- TGG TTA GTT TCT TTT CCT CCG C -′3), resulting in a 500 bp fragment, which is used as genetic markers for rumen fluke species (Itagaki et al. 2003). The reaction was carried out in a volume of 50 µl, containing 2 µl of DNA template and a final concentration of 1 × GoTaq® green Mastermix (Promega, USA), 0.2 mM dNTPs, 2 mM MgCl_2_, 0.4 µM per primer, and 1.25 U of GoTaq® G2 DNA polymerase (Promega, USA). Thermocycling conditions were as follows: initial denaturation at 94 °C for 2 min followed by 35 cycles of 94 °C for 1 min, 55 °C for 90 s, and 72 °C for 1 min. A final elongation at 72 °C for 10 min ended the program.

A 641 bp section of the mitochondrial *COI* section was amplified with Neodermata-specific primers (COI_Neod_FW_5´- TTT ACT TTG GAT CAT AAG CG -3´ and COI_Neod_Rv 5´- CCA AAA AAC CAA AAC ATA TGT TGA A -3´ (Duscher et al. 2015)). The reaction was set up in a total volume of 50 µl containing 2 µl of DNA template and a final concentration of 1 × GoTaq® green Mastermix (Promega, USA), 0.2 mM dNTPs, 0.8 µM per primer, and 1.25 U of GoTaq® G2 DNA polymerase (Promega, USA). Thermocycling conditions were as follows: initial denaturation at 95 °C for 2 min followed by 35 cycles of 95 °C for 1 min, 48 °C for 1 min, and 72 °C for 1 min. A final elongation at 72 °C for 5 min ended the program.

All amplicons were checked on 1% agarose gel stained with Midori Green Advance DNA stain (Nippon Genetics Europe, Germany), and purification and bi-directional sequencing were performed at Microsynth AG, Switzerland.

The sequences were analyzed using Bioedit v.7.0.5.3 (Hall 1999). BLAST searches were performed on NCBI GenBank (http://www.ncbi.nlm.nih.gov/BLAST) to gather all *COI* and *ITS2* sequences of the family Paramphistomidae. The sequences were combined with the new data and the sequences were collapsed before the phylogenetic analysis. Model tests were performed for both data sets using the IQ-tree webserver (http://iqtree.cibiv.univie.ac.at/ (Trifinopoulos et al. 2016)). Maximum likelihood (ML) trees were calculated using IQ-tree (Minh et al. 2020) with the substitution models K2P + I + G4 for ITS2 and GTR + F + G4 for *COI* sequences. Bayesian Inference (BI) trees were calculated with MrBayes v.3.1.2 (Huelsenbeck and Ronquist 2001), applying the substitution model GTR + G + I. The Bayesian analyses were run for 5^10^ generations (two runs each with four chains, one of which was heated), sampling every 100th tree. The first 25% of trees were discarded as burn-in, and a 50% majority rule consensus tree was calculated from the remaining 37,500 trees. The nodes in the trees are provided with both BI posterior probabilities and ML bootstrap values.

## Results and discussion

The fluke burden of the 15 dairy cows ranged from one to 536 (median 14); the eight animals from the cow-calf operations harbored 56 to 1231 rumen flukes (median 278). Neither the owners of the animals nor the responsible veterinarians reported clinical signs in the animals that could be related to the infection. The higher burden in the animals from the cow-calf operations compared to the dairy cows may indicate that production systems relying extensively on grazing are likely to favor larger paramphistome burdens. Similar findings with respect to the specific importance of rumen fluke infections for animals from cow-calf operations were recently reported from the Netherlands and Germany based on coproscopical surveys (Ploeger et al. 2017; Forstmaier et al. 2021). The rumen fluke counts fit within the wide range of counts reported from other countries in Europe, i.e., Belgium, France, the Netherlands, and Spain (Szmidt-Adjidé et al. 2000; González-Warleta et al. 2013; Ferreras et al. 2014; Malrait et al. 2015; Ploeger et al. 2017).

By sequencing of a section of the mitochondrial *COI*, the common DNA barcode sequence, and the complete nuclear *ITS2* sequence of the 35 flukes, six specimens were identified as *P. leydeni* and 29 specimens as *C. daubneyi*. The *P. leydeni* flukes originated from five dairy cows from three farms in Bavaria harboring between one and four flukes. The *C. daubneyi* flukes were diagnosed in ten dairy cows from three farms (Bavaria, Saxony, Tyrol) and the eight animals from cow-calf operations with fluke counts ranging between two and 536 and 56 and 1231, respectively.

**Fig. 1:**
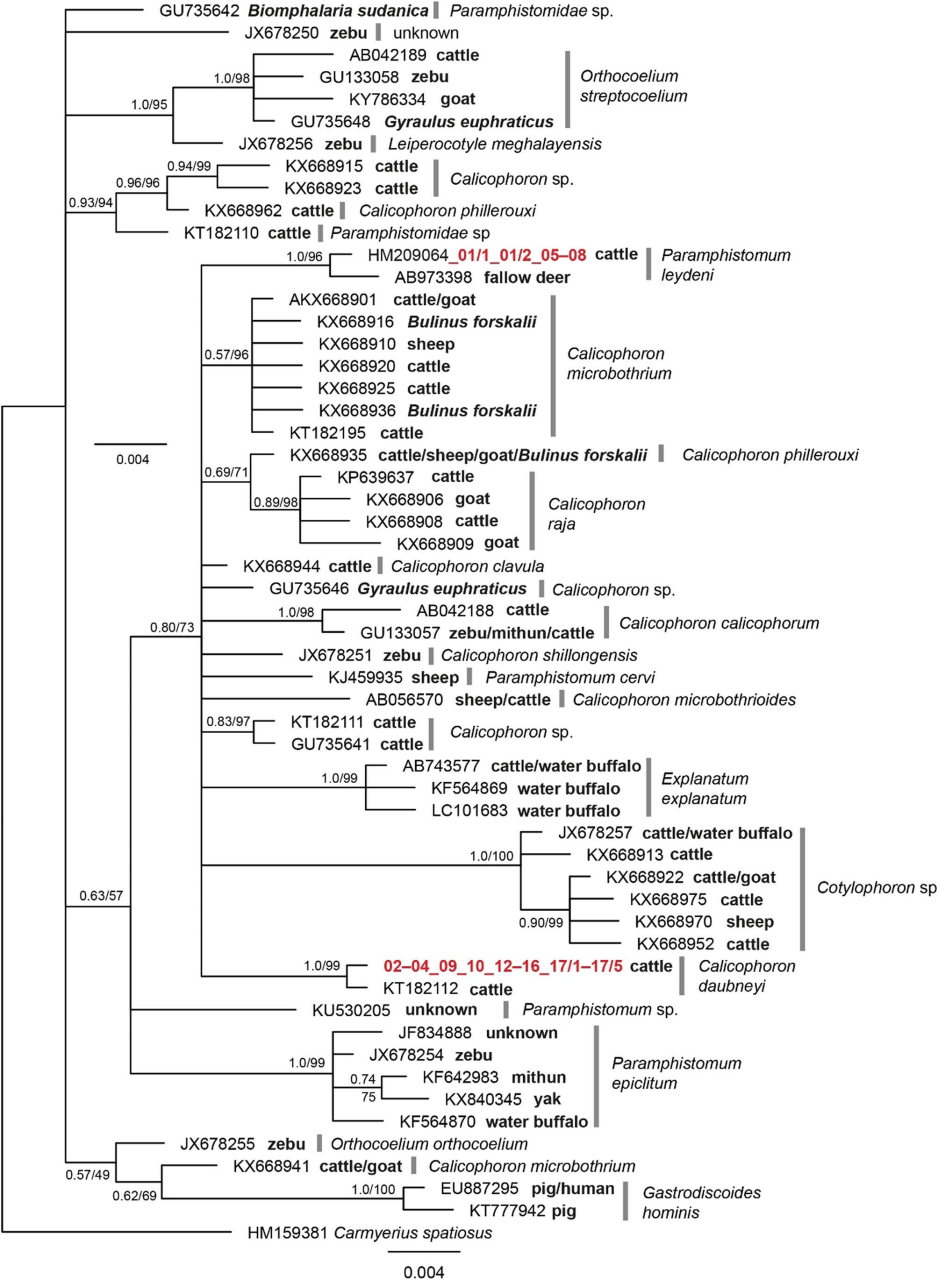
Bayesian haplotype tree of *ITS2* sequences of Paramphistomidae. Sequences obtained within this study are marked in red and belong to the species *C. daubneyi* and *P. leydeni*, revealing one haplotype for both species. Nodes are marked with BI posterior probabilities and ML bootstrap values

**Fig. 2:**
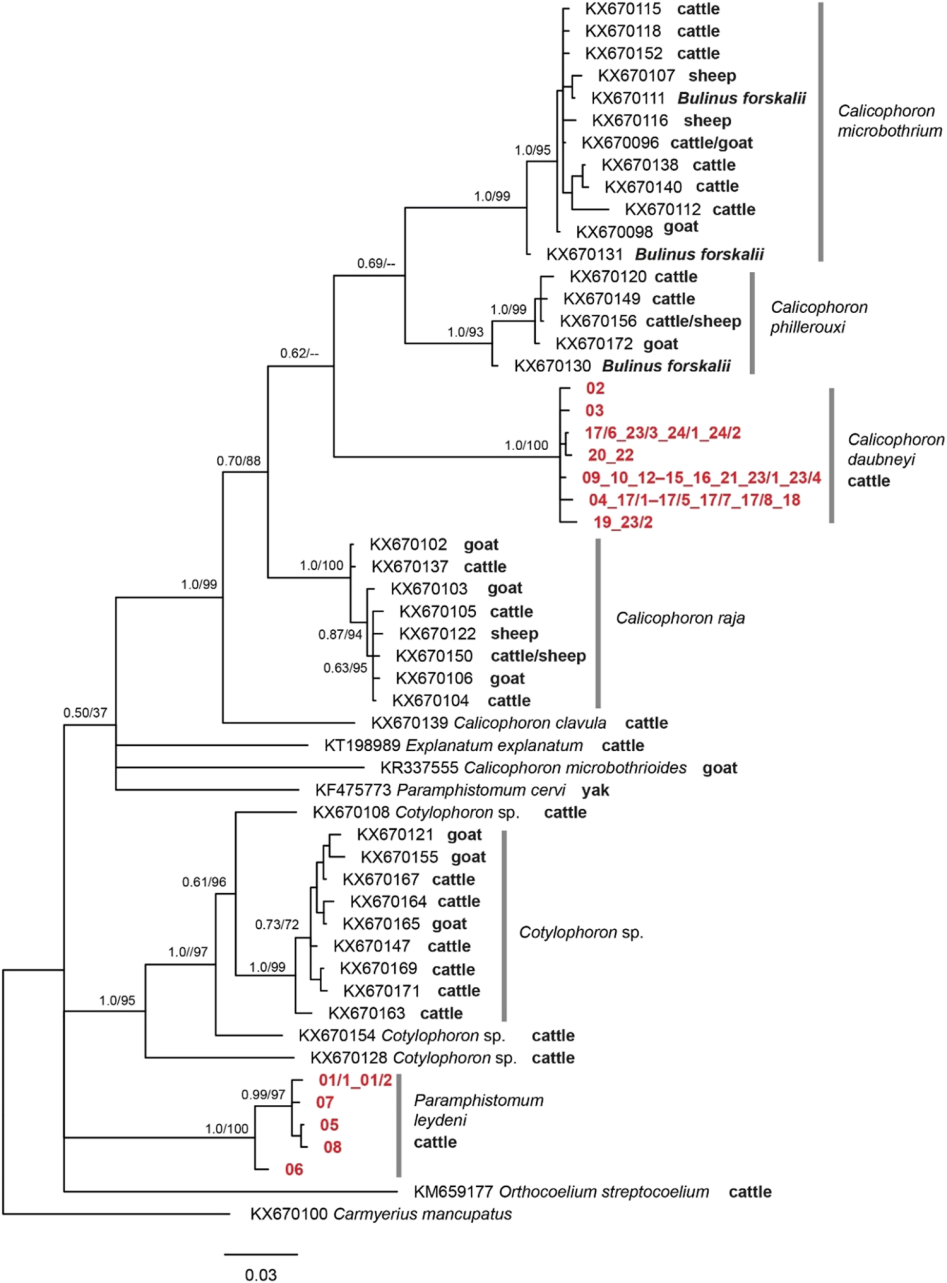
Bayesian haplotype tree of *COI* sequences of Paramphistomidae, sequences obtained within this study are marked in red. Both *C. daubneyi* and *P. leydeni* featured several similar *COI* lineages. Nodes are marked with BI posterior probabilities and ML bootstrap values

The flukes featured identical *ITS2* sequences within the species and several similar *COI* lineages. The *COI* sequences represent the first DNA-barcodes generated for *P. leydeni* and *C. daubneyi*. Bayesian trees of *ITS2* and *COI* sequences were calculated (Figs. 1 and 2), and the sequences were deposited in GenBank® (Accession numbers *COI*: MZ519977–MZ520011; accession numbers *ITS2*: MZ532797–MZ532831). The *ITS2* region was used to determine the species affiliation, which proved to be a convenient marker within this group, since it shows inter-specific variation between species of the genus *Calicophoron* but no or little intra-specific variation (Rinaldi et al. 2005). The *COI* sequences are the first ones reported for the two species. Hence, the obtained sequences contribute to the molecular identification of paramphistosomes and provide an important basis for future studies.

This molecular investigation reveals the first record of *C. daubneyi* in Austria and it adds evidence for a common occurrence of this species in Germany where *C. daubneyi* was identified initially in the late 1970s in eastern Germany based on histomorphology of adult flukes (Odening et al. 1978) and more recently several times using molecular identification of fluke eggs (May et al. 2019; Wenzel et al. 2019; Forstmaier et al. 2021). The findings of this investigation support that *C. daubneyi* is currently apparently the most widespread species of rumen flukes of domestic ruminants in Europe (Wenzel et al. 2019; Forbes 2021). While a just published paper reported the first molecular identification of *P. leydeni* fluke eggs in bovine feces in Germany (Forstmaier et al. 2021), the present investigation features the first molecular proof for the occurrence of *P. leydeni* in Germany based on the examination of adult flukes isolated from cattle. In the past, the histomorphological examination of fluke specimens of bovine origin from Germany led to a controversial interpretation with respect to the identification of *P. leydeni*, which was considered synonymous with *P. cervi* by some authors (Odening et al. 1978; Eduardo 1982; Odening 1983). Using molecular diagnosis, *P. leydeni* has been identified parasitizing cervids in Europe in the recent past, including one case of co-infection with *P. cervi* (O’Toole et al. 2014; Sindičić et al. 2017), but was occasionally identified from fluke eggs excreted by sheep and cattle in Ireland and the Netherlands, respectively (Martinez-Ibeas et al. 2016; Ploeger et al. 2017).

This investigation was based on an opportunistic sample collection and was of limited extent in regard to the number of flukes examined in total and as to the portion of flukes examined per animal, in particular for the animals which were demonstrated to harbor *C. daubneyi*. Therefore, the occurrence of flukes of species other than *C. daubneyi* in the animals harboring *C. daubneyi* cannot be ruled out. However, to the best knowledge of the authors, no mixed species rumen fluke infections have been reported from domestic ruminants in Europe based on molecular diagnosis. Thus, further studies should be conducted to add knowledge on the diversity of species of rumen flukes parasitizing both domestic and wild ruminants in Europe as well as to document their prevalence and the parasite burden.

## Declarations

### Disclaimer

This document is provided for scientific purposes only. Any reference to a brand or trademark herein is for informational purposes only and is not intended for a commercial purpose or to dilute the rights of the respective owner(s) of the brand(s) or trademark(s).
